# Effects of dexamethasone on post-operative cognitive dysfunction and delirium in adults following general anaesthesia: a meta-analysis of randomised controlled trials

**DOI:** 10.1186/s12871-019-0783-x

**Published:** 2019-06-29

**Authors:** Li-Qin Li, Cong Wang, Mei-Dan Fang, Hong-Yu Xu, Hong-Liu Lu, Hou-Zhong Zhang

**Affiliations:** Department of Anesthesiology, Jilin University Second Hospital, No. 218 Ziqiang street, Changchun, Jilin, 130021 People’s Republic of China

## Abstract

**Background:**

Several studies have investigated the effects of dexamethasone on post-operative cognitive dysfunction (POCD) or post-operative delirium (POD); however, their conclusions have been inconsistent. Thus, we conducted a meta-analysis to determine the effects of dexamethasone on POCD and POD in adults following general anaesthesia.

**Methods:**

The Cochrane Central Register of Controlled Trials (2018, Issue 11 of 12) in the Cochrane Library (searched 17 November 2018), MEDLINE OvidSP (1946 to 16 November 2018) and Embase OvidSP (1974 to 16 November 2018) were searched for randomised controlled trials that evaluated the incidence of POCD and POD following dexamethasone administration in adults (age ≥ 18 years) under general anaesthesia. We used the Grading of Recommendations, Assessment, Development and Evaluations framework to assess the quality of the evidence.

**Results:**

Five studies were included (three studies with 855 participants in the dexamethasone group and 538 participants in the placebo group for the incidence of POCD, and two studies with 410 participants in the dexamethasone group and 420 participants in the placebo group for the incidence of POD). There was no significant difference between the dexamethasone group and the placebo group in terms of the incidence of POCD 30 days after surgery (RR [relative risk] 1.00; 95% CI [confidence interval: 0.51, 1.96], *P* = 1.00, *I*^2^ = 77%) or the incidence of POD (RR 0.96; 95% CI [0.68, 1.35], *P* = 0.80, *I*^2^ = 0%). However, both analyses had some limitations because of limited evidence and clinical heterogeneity, and we considered the quality of the evidence for the post-operative incidence of POCD and POD to be very low.

**Conclusions:**

This meta-analysis revealed that prophylactic dexamethasone did not reduce the incidence of POCD and POD. Trials of alternative preventive strategies for POCD and POD, as well as a better understanding of the pathophysiology of those complex syndromes, are still needed to make progress in this field.

**Trial registrationr:**

This study is registered with PROSPERO, 23 October 2018, number CRD42018114552. Available from https://www.crd.york.ac.uk/PROSPERO/#recordDetails.

**Electronic supplementary material:**

The online version of this article (10.1186/s12871-019-0783-x) contains supplementary material, which is available to authorized users.

## Background

Post-operative cognitive dysfunction (POCD) and post-operative delirium (POD) are neuropsychological disorders that can occur following the administration of general anaesthesia. POCD is one of the most common complications in both young and elderly patients [1]. Evered et al. reported that the incidence rates of POCD at 7 days post-surgery were 17% for total hip joint replacement surgery and 43% for coronary artery bypass graft surgery. The incidence of POCD at 3 months post-surgery for both groups combined was 17% [2]. POCD is subtle and can only be detected by several neuropsychological tests, which are performed before and after surgery [3]. POD is a transient disturbance of a patient’s consciousness, attention, cognition and perception, which can last from a few hours to a few days and can fluctuate in severity [4]. POCD and POD are serious complications that are associated with prolonged length of hospital stay, delayed recovery of function, decreased quality of life and increased risk of further complications and mortality [5–7].

Unfortunately, there are still many gaps in our knowledge regarding the pathophysiology of POCD and POD, which hinder our prevention and treatment attempts. At present, the prevention of POCD and POD is mainly based on non-pharmacological measures and comprehensive geriatric assessments focusing on risk factors [8, 9]. But the high prevalence of POCD and POD persists despite these attempts, and given the lack of human resources in hospitals, the idea that medication could prevent POCD and POD is interesting and potentially time-saving. There is growing evidence that the brain’s reaction to a peripheral inflammatory process may play a role in the development of POD and POCD. Dillon et al. found that elevated preoperative and post-operative levels of C-reactive protein (CRP) are associated with POD [10]. A recent meta-analysis has suggested that high concentrations of inflammatory markers in the peripheral circulation and cerebrospinal fluid are associated with POCD and POD [11]. Dexamethasone is a long-acting glucocorticoid widely used in various inflammatory diseases [12, 13]. If the inflammatory response plays a role in the occurrence of POCD and POD, then inhibiting the inflammatory response through dexamethasone may prevent POCD and POD. In addition, several studies have investigated the effects of dexamethasone on POCD and POD; however, their conclusions have been inconsistent. Therefore, we have conducted a meta-analysis to evaluate the effects of prophylactic dexamethasone administration on the incidence of POCD and POD. Although the focus was on older adults after surgery, we were concerned that the existing study was too limited; hence, the search included all adults under general anaesthesia.

## Methods

We adhered to the Preferred Reporting Items for Systematic Reviews and Meta-Analyses (PRISMA) statement [14], and we used the Grading of Recommendations, Assessment, Development and Evaluations (GRADE) framework to assess the quality of the evidence [15]. This study is registered with PROSPERO, 23 October 2018, number CRD42018114552.

### Eligibility and exclusion criteria

We selected all of the studies that met the following eligibility criteria: (1) randomised controlled trials; (2) adults (≥18 years old) who underwent general anaesthesia; (3) perioperative administration of intravenous dexamethasone in order to prevent POCD and POD (including administration during the preoperative, intraoperative and post-operative periods) versus no interventions (no drug administered or placebo group), regardless of the dose administered; (4) the incidence of POCD and POD as a primary or secondary outcome and (5) availability of the full text in English. We excluded studies in which administration of another drug was used in the control group, dexamethasone was administered by another route, and no assessment tools were available to evaluate the incidence of POCD and POD.

### Search strategy

We performed a systematic search of the Cochrane Central Register of Controlled Trials (2018, Issue 11 of 12) in the Cochrane Library (searched 17 November 2018), MEDLINE OvidSP (1946 to 16 November 2018) and Embase OvidSP (1974 to 16 November 2018). The search strategy is shown in Additional file 1: Appendix. We also manually searched the references of the included studies and reviews for additional studies. The following sources of ongoing and unpublished trials were screened: http://www.controlled-trials.com and http://www.clinicaltrials.gov.

### Endpoints

The primary outcomes are the incidence of POCD or POD according to the author’s own definition; however, there is a need for an objective assessment tool for POCD and POD.

The secondary outcomes are as follows: (1) all-cause mortality at 30 days, (2) any post-operative complications, (3) level of C-reactive protein (CRP) measured within the first 24 h post-operatively and (4) hospitalisation (measured in days) and intensive care unit (ICU; measured in hours) duration.

### Study selection

After importing the search results into EndNote X9, two review authors (Li and Wang) independently screened the reports according to the predetermined inclusion criteria. Firstly, duplicate reports were removed, and the studies were selected on the basis of the title and abstract. Subsequently, the full text was screened for compliance with the inclusion criteria. A disagreement between Li and Wang was resolved through discussion and consensus with a third reviewer (Fang).

### Extraction of data and assessment of risk of Bias in the included studies

Li and Wang extracted data independently from eligible studies using a predesigned form. A disagreement between the two review authors was resolved through discussion and consensus with a third reviewer (Fang). Two review authors (Li and Wang) independently assessed the risk of bias for each included study using the Cochrane Collaboration’s tool [16]. We assessed each study according to the following seven domains: random sequence generation, allocation concealment, participant and personnel blinding, outcome assessment blinding, incomplete outcome data, selective reporting and other biases. We rated the overall risk of bias of a study as low if all of the domains were ‘low risk’, high if one or more of the domains were identified as ‘high risk’ and unclear if one or more of the domains were identified as ‘unclear risk’.

### Synthesis of results

We used the Cochrane Review Manager 5 for statistical analysis of the data. Only primary and secondary outcomes that we have defined in advance were used in our analysis. For those studies that did not report a mean and standard deviation (SD), we did not hand and transformate the date, because we did not know if it was normally distributed [17, 18]. Dichotomous variable data (e.g. POCD and POD incidence, all-cause mortality at 30 days and any post-operative complications) were expressed as relative risk (RR) with 95% confidence intervals (CIs), whereas continuous data (e.g. the level of CRP and length of hospital and ICU stay) were reported as weighted mean differences (WMD) with 95% CI.

### Assessment of heterogeneity and data synthesis

We used the chi-square test and calculated the I2 statistic to assess the heterogeneity of the studies, and considered heterogeneity to be low (I2 < 50%), moderate (I2 = 50 to 75%) and high (I2 = 75 to 100%). We performed a subgroup analysis of cardiac surgery versus non-cardiac surgery and low-dose (≤0.2 mg/kg) versus high-dose (> 0.2 mg/kg) dexamethasone to explore clinical heterogeneity. We expected substantial clinical and methodological heterogeneity, so we used the randomised effect model [19]. A *P*-value < 0.05 was considered as statistically significant. If it was inappropriate to undertake the meta-analysis, we instead carried out a descriptive analysis of the study.

### Assessment of publication Bias and sensitivity analysis

Where the number of included studies was more than 10, we assessed the risk of publication bias among the included studies based on a funnel plot. We performed the following sensitivity analysis to assess the stability of POCD and POD: (1) excluding reports with a high risk of bias and (2) using different models (the randomised effect model and the fixed effect model).

## Results

### Study selection

**Fig. 1:**
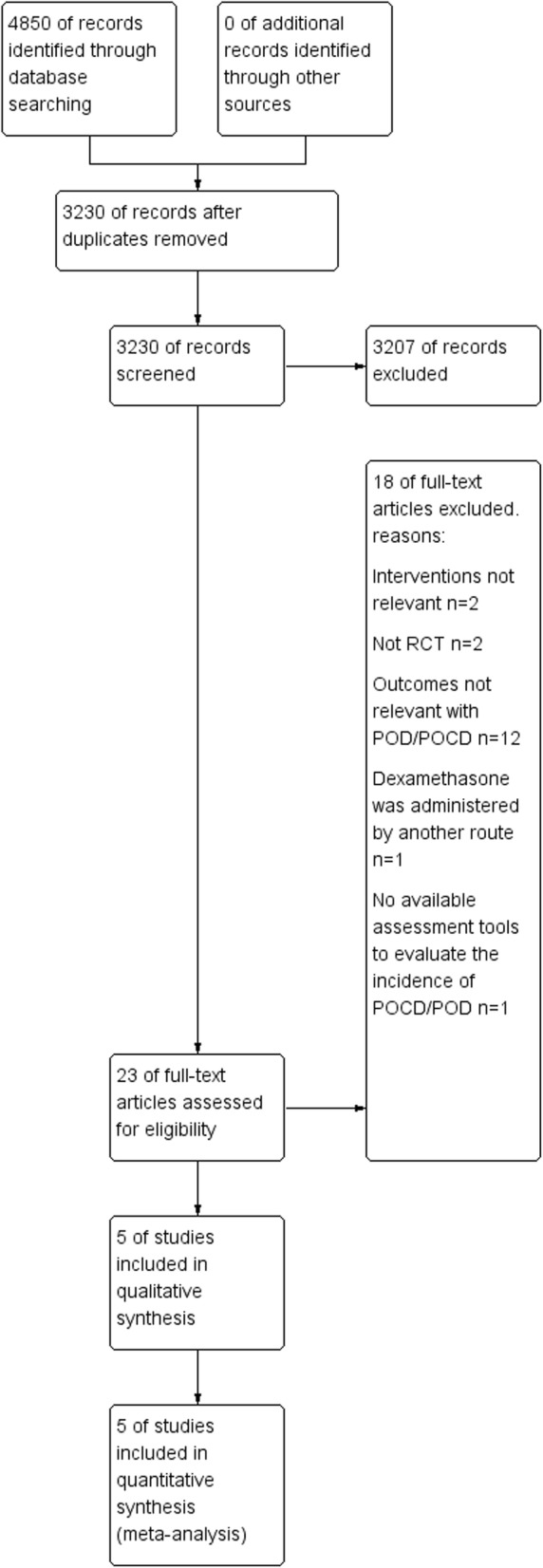
Study flow diagram

Details of the flow of retrieved results and study selection are shown in Fig. 1. According to the predefined search strategy, we retrieved 4850 studies. After removing the duplicate studies, we screened the remaining 3230 studies based on the title and abstract. We then read the full text of 23 studies for further assessment according to the inclusion criteria. No eligible studies were found by manual retrieval. Finally, five studies were included in our analysis. After searching the clinical trials registration platform, we identified two ongoing studies that will be assessed after they are completed [20, 21].

### Study characteristics

Table 1 shows the characteristics of the included studies. Four of the included studies involved cardiac surgery [22–25], whereas the remaining one involved microvascular decompression surgery [26]. A larger, multicentre placebo-controlled randomised clinical trial was excluded because it did not report the occurrence of POCD, and not all patients had been assessed by the available delirium assessment tools [27]. The mean age of all the participants in the included studies was over 60 years, except for one study [26]. Four studies used a two-arm design in comparing dexamethasone with placebo except one, which used a three-arm design [26]. Three studies assessed the effects of dexamethasone on POCD [22, 24, 26], whereas two studies investigated the effects of dexamethasone on POD [23, 25]. Dexamethasone doses and administration time varied, as shown in Table 2. The definition and assessment tools for POCD and POD were also different (Table 2).

**Table 1:** The characteristics of the included studies

Studies ID	Design	Country	Age (min ± SD)		M/F		Type of surgery	Outcome
			Dex	Control	Dex	Control		
Fang 2014	3-arm RCT	China	48.9± 5.35^a^	48.0 ± 5.77	247/388	121/198	microvascular decompression	POCD
Glumac 2017	2-arm RCT	Croatia	63.7± 9.0	64.2± 9.4	63/22	64/20	Cardiac surgery	POCD
Mardani 2013	2-arm RCT	Iran	64.5± 11.10	60.04± 12.77	36/7	44/6	Cardiac surgery	POD
Ottens 2014^b^	2-arm RCT	The Netherla-nds	63.4± 12.3	65.4± 1.5	103/37	109/29	Cardiac surgery	POCD
Sauer 2014^b^	2-arm RCT	The Netherla-nds	67 ± 12	66 ± 12	255/112	225/145	Cardiac surgery	POD

*SD* = standard deviation, *POCD* = postoperative cognitive dysfunction, *POD* = postoperative delirium, *Dex* = dexamethasone. ^a^This 3-arm RCT reported the age was 48.9 ± 5.35, 48.0 ± 5.60 in dexamethasone 0.1 mg/kg and dexamethasone 0.2 mg/kg respectively. ^b^Ottens 2014 and Sauer 2014 were two substudies of a larger, multicenter placebo-controlled randomized clinical trial

**Table 2:** Characteristics of intervention and outcomes

Study ID	Dexamethas one doses	Control	Time of intervention or control	POCD/POD assessment method	POCD/POD definition	Assessment time
Fang 2014	D1:0.1 mg/kg D2:0.2 mg/kg	NS	before induction of anesthesia	Neuro-Psychologic-al test battery	An individual whose postoperative performance deteriorated by 1 or more SDs on 2 or more tests was classified as having experienced early POCD.	the day before and On the fifth postoperativ-e day
Glumac 2017	0.1 mg/kg	NS	10 h before the surgery	a battery of five neuropsychological tests	Authous calculated the Jacobson and Truax Reliable Change Index (RCI) for each patient in the dexamethasone and placebo groups. POCD in an individual is defined as an RCI equal to or less than S1.96 on at least one test	on the 6th day after the surgical procedure
Mardani 2013	8 mg DEX intravenous before induction of anesthesia followed by 8 mg every 8 h for 3 day.	NS	before induction of anesthesia followed every 8 h for 3 day	MMSE	delirium disorder was diagnosed if DSM-IV criteria were met in a patient	Preoperative day (PROD), first, second, andthird postoperativ-e day.
Ottens 2014	1 mg/kg (maximum 100 mg)	NS	shortly after induction of general anesthesia	a battery of five neuropsychological tests	Authous calculated the Jacobson and Truax Reliable Change Index (RCI), they defined POCD in an individual patient as an RCI equal to or less than −1.96, or Z-score equal to or less than − 1.96 in at least two different tests.	1 day before surgery and 1 month and 12 months after surgery
Sauer 2014	1 mg/kg (maximum 100 mg)	NS	at the time of induction of anesthesia	CAM-ICU	POD: diagnosed by the Confusion Assessment Method (CAM) adapted for the ICU (CAM-ICU) .	The primary study outcome was the presence of delirium on any of the first 4 postoperativ-e days. 7 days a week at a fixed time point

*POCD* = postoperative cognitive dysfunction, *POD* = postoperative delirium, *NS*: normal saline, *MSSE* = Mini-Mental State Examination, *SD* = standard deviation, *RCI* = Jacobson and Truax Reliable Change Index, *CAM* = Confusion Assessment Method, *CAM-ICU*=Confusion Assessment Method adapted for the ICU, *PROD* = Preoperative day

### Risk of Bias in the included trials

**Fig. 2:**
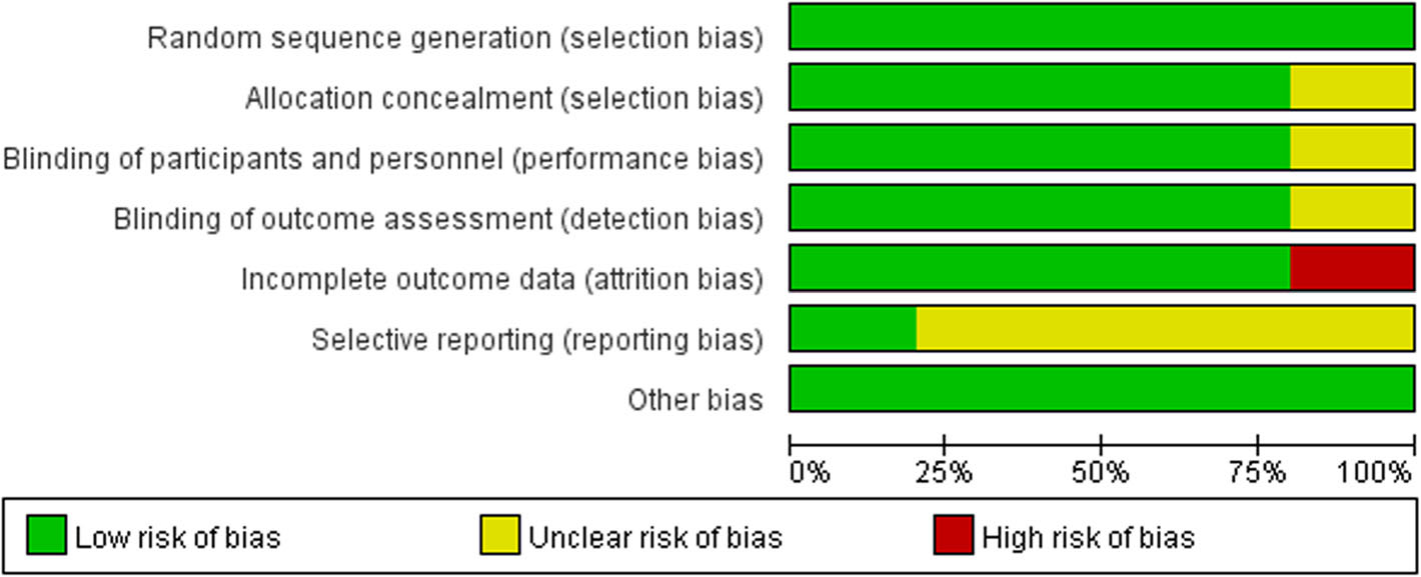
Risk of bias graph. Review authors’ judgements about each risk of bias item presented as percentages across all included studies

**Fig. 3:**
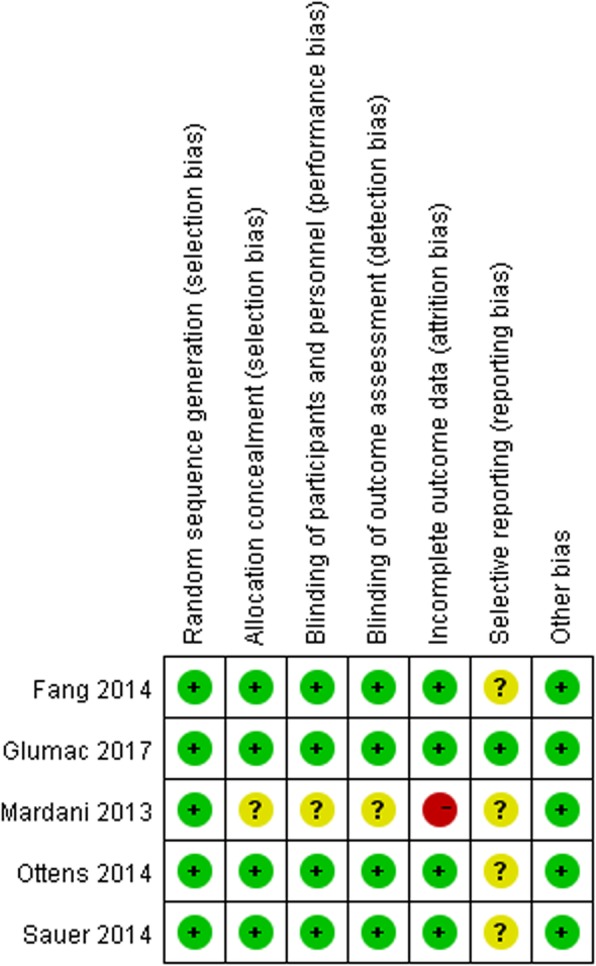
Risk of bias summary: review authors’ judgements about each risk of bias item for each included study

The risk of bias in the included studies is summarised in Figs. 2 and 3. One study was identified as ‘low risk’ in all domains [22], three studies had an unclear risk of bias in one of the seven domains because of reporting bias [24–26] and one study had a high risk of bias in one of the seven domains [23].

### Incidence of POCD

**Fig. 4:**
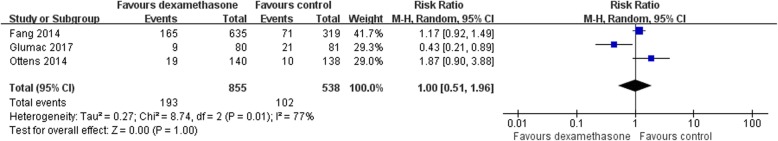
Forest plot of comparison: Dexamethasone vs Control, Outcome: POCD in 30 days after surgery

Three studies reported the incidence of POCD, in which data were reported as the number of participants [22, 24, 26]. Fang et al. reported the incidence of POCD at 5 days post-operatively [26], and Glumac et al. at 6 days post-operatively [22]. Ottens et al. assessed POCD at 1 and 12 months post-operatively [24], but we only selected the results reported 1 month after surgery in order to avoid double counting. Fang et al. used neurocognitive tests to assess the incidence of POCD, whereas Glumac et al. and Ottens et al. both used a battery of five neuropsychological tests, making their definitions of POCD different. This meta-analysis included 855 participants in the dexamethasone group and 538 participants in the placebo group, which accounted for 95% of the total 1460 enrolled participants. There was no significant difference in the incidence of POCD 30 days after surgery between the dexamethasone group and the control group (RR 1.00; 95% CI [0.51, 1.96], *P* = 1.00, *I*^2^ = 77%; Fig. 4). We judged, the quality of the evidence to be very low based on the GRADE framework: (1) two studies had a unclear risk of bias; (2) the definitions of the outcome and the assessment tools were different; (3) results were inconsistent and (4) the result was imprecise.

### Incidence of POD

**Fig. 5:**
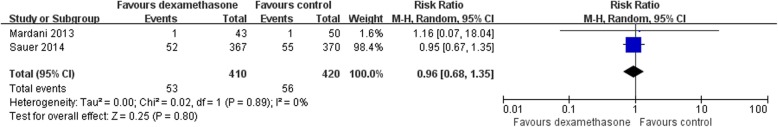
Forest plot of comparison: Dexamethasone vs Control, Outcome: POD

Two studies reported the incidence of POD, in which the data were reported as the number of participants [23, 25]. Mardani et al. reported the incidence of POD on the preoperative day, followed by the first, second and third post-operative days. In order to avoid double counting and given that POD typically occurs 2 to 3 days after surgery, we only selected the results reported on the third post-operative day [23]. Sauer et al. reported the incidence of the first four post-operative days [25]. Mardani et al. used the Mini-Mental State Examination (MMSE) to assess POD [23], whereas Sauer et al. used the Confusion Assessment Method (CAM) adapted for the ICU (CAM-ICU) [25]. The meta-analysis showed that there was no significant difference in the incidence of POD between the dexamethasone and placebo groups (RR 0.96; 95% CI [0.68, 1.35], *P* = 0.80, *I*^2^ = 0%; Fig. 5). We deemed the quality of the evidence to be very low because (1) the two studies had an ‘unclear risk’ of reporting bias and (2) the result was imprecise.

### Secondary outcomes

No studies reported the all-cause mortality at 30 days post-surgery. One of the five included studies reported post-operative complications including deep sternal wound infection, leg infection, sepsis and pneumonia in addition to cardiac, cerebrovascular, respiratory and renal complications [23]. The study showed that there was no significant difference between the dexamethasone and control groups in post-operative complication rates [23]. We consider the quality of the evidence for this outcome to be very low because of the limited evidence available and the fact that the study was at high risk of bias.

Only one study reported the level of CRP [22], which was measured 12 h after surgery and on each of the first three post-operative days. The level of CRP in the dexamethasone group was lower compared with those in the placebo group at all-time points (*P* < 0.001). We deemed the evidence for this outcome to be of very low quality based on the limited evidence available.

**Fig. 6:**
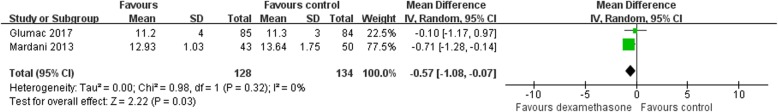
Forest plot of comparison: Dexamethasone vs Control, Outcome: Duration of hospitalization (measured in days)

Two studies reported the duration of hospitalisation (measured in days) [22, 23]. Our meta-analysis showed that the use of dexamethasone reduced the length of hospital stay (WMD − 0.57 days; 95% CI [− 1.08, − 0.07], *P* = 0.03, *I*^2^ = 0%; Fig. 6), but the difference was so small that it did not have a clinical significance. We considered the quality of the evidence for this outcome to be very low because of the limited availability of evidence and imprecision of the result.

**Fig. 7:**
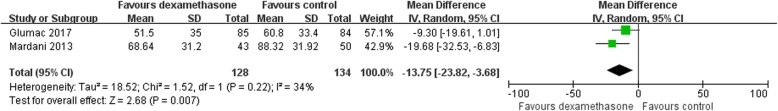
Forest plot of comparison: Dexamethasone vs Control, Outcome: Length of ICU stay (measured in hours)

Three studies reported the length of ICU stay [22, 23, 25]. Mardani et al. and Glumac et al. reported the duration as mean ± SD [22, 23]. Sauer et al. reported the duration as median (interquartile range); this was 23 (20–24) h in the dexamethasone group and 22 (20–24) h in the control group [25]. We excluded Sauer et al. and conducted a meta-analysis showing that the use of dexamethasone reduced the length of ICU stay (WMD − 13.75 h, 95% CI [− 23.82, − 3.68], *P* = 0.007, *I*^2^ = 34%; Fig. 7). We deemed the quality of the evidence for this outcome to be very low because of the limited availability of evidence and imprecision of the result.

### Subgroup analysis

**Fig. 8:**
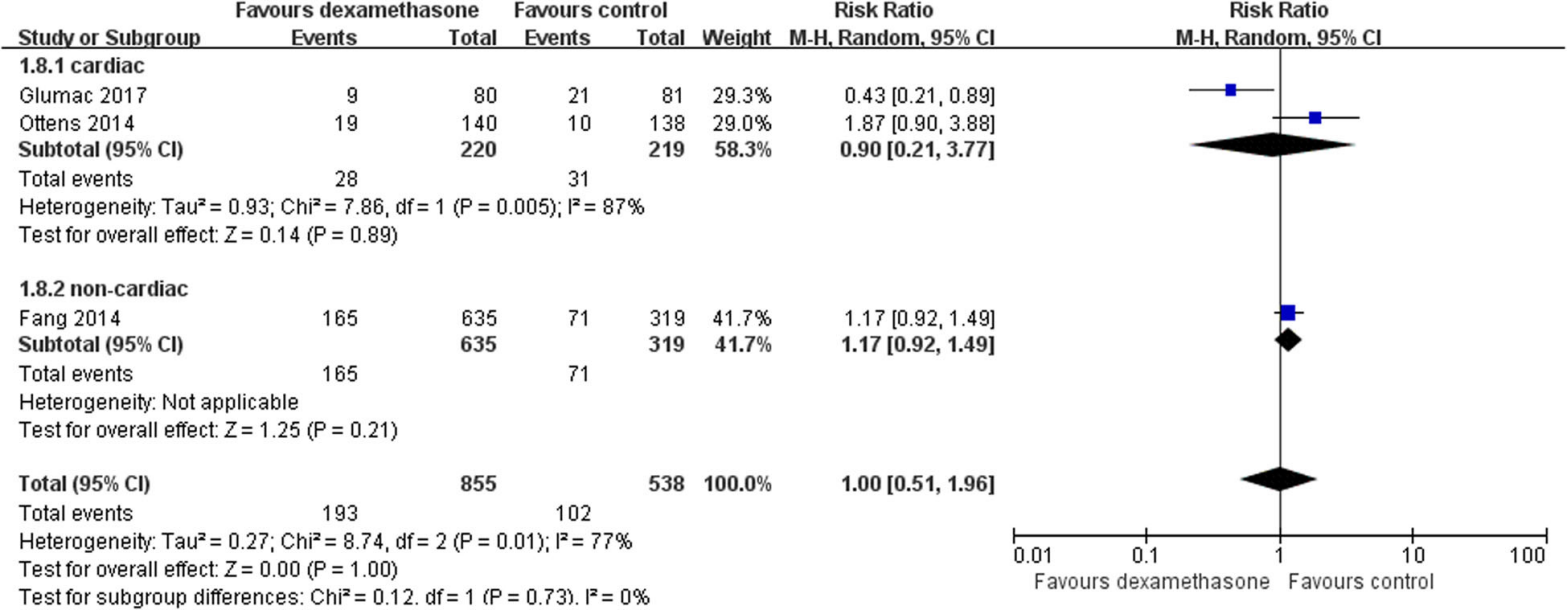
Forest plot of comparison: Dexamethasone vs Control, Outcome: Subgroup analysis of cardiac surgery versus noncardiac surgery

For POCD, one study described non-cardiac surgery [26], whereas two studies described cardiac surgery [22, 24]. We performed a subgroup analysis of cardiac surgery versus non-cardiac surgery (as a subgroup of cardiac surgery only). We noted no difference in the incidence of POCD between the dexamethasone and control groups when non-cardiac surgery was excluded (RR 0.90, 95% CI [0.21, 3.77], *P* = 0.89, *I*^2^ = 87%; 439 participants, Fig. 8). We also found no significant difference between the subgroups (*P* = 0.73) as shown in Fig. 8. For POD, the participants of all the studies underwent cardiac surgery.

**Fig. 9:**
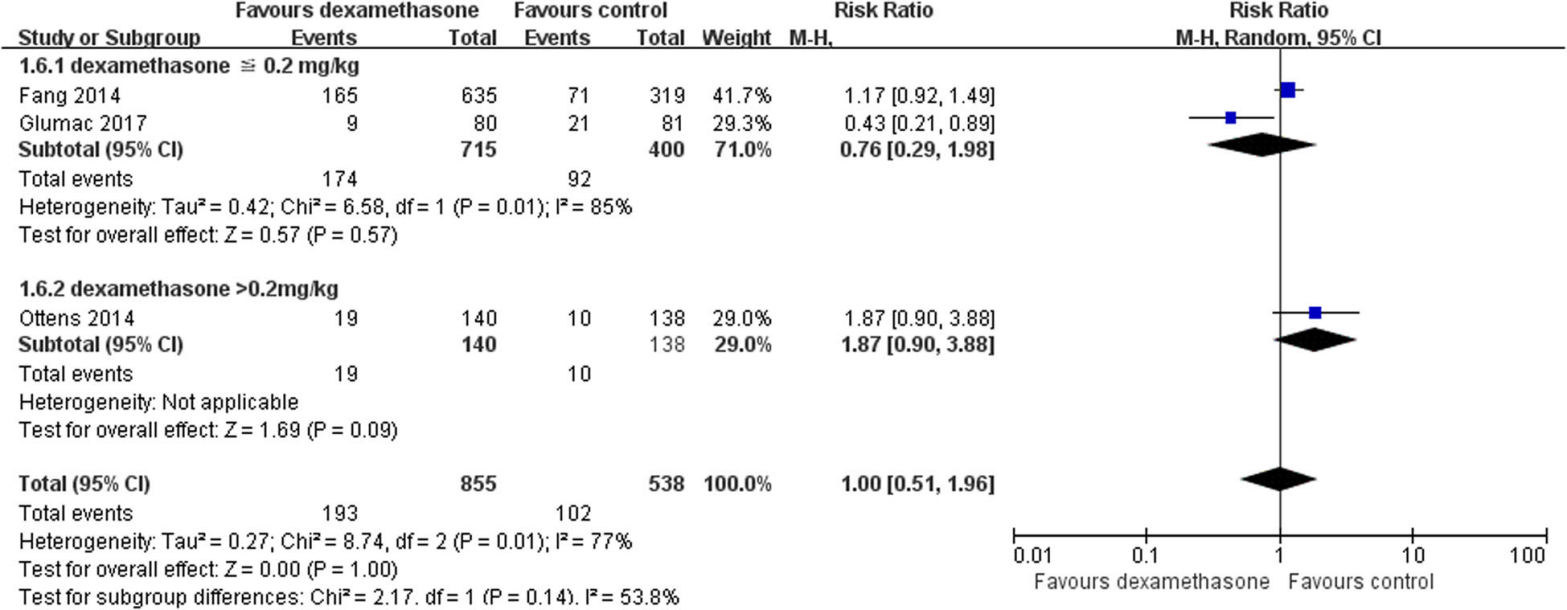
Forest plot of comparison: Dexamethasone vs Control, Outcome: Subgroup analysis of low dose (≦ 0.2 mg/kg) versus high dose (> 0.2 mg/kg) dexamethasone

For POCD, two studies administered a low-dose of dexamethasone (≤0.2 mg/kg) [22, 26], and one study administered a dexamethasone dose of 1 mg/kg (maximum 100 mg) [24]. We also performed a subgroup analysis of low-dose (≤0.2 mg/kg) versus high-dose (> 0.2 mg/kg) dexamethasone (as a subgroup of low-dose ≤0.2 mg/kg only). This subgroup analysis showed no significant difference in the incidence of POCD between the low-dose dexamethasone (≤0.2 mg/kg) and control groups (RR 0.76, 95% CI [0.29, 1.98]; 1115 participants, Fig. 9). We noted no significant difference between the subgroups (*P* = 0.14) as shown in Fig. 9. For POD, one study administered 8 mg of dexamethasone before the induction of general anaesthesia and then followed by another 8 mg every 8 h [23]. In another study, dexamethasone was administered at a dose of 1 mg/kg (maximum 100 mg) [25]; thus, we did not conduct a subgroup analysis.

### Assessment of publication Bias and sensitivity analysis

Considering that the number of the included studies was small, we did not conduct an assessment of publication bias. Based on the prior definition, there was only one study with a low risk of bias, so we did not conduct the sensitivity analysis based on the risk of bias. Sensitivity analyses using the fixed effect model yielded stable overall results: (POCD: RR 1.10, 95% CI [0.89, 1.37]; POD: RR 0.96, 95% CI [0.68, 1.35]).

## Discussion

This meta-analysis aimed to assess the effects of dexamethasone on POCD and POD in adults following general anaesthesia. We found that prophylactic intravenous administration of dexamethasone did not reduce the incidence of POCD 30 days following surgery or POD. Furthermore, our meta-analysis showed that the use of dexamethasone reduced the length of hospital stay, but the difference was so small that it did not have a clinical significance. Nevertheless, the results of our meta-analysis suggested that the length of ICU stay was shorter in the dexamethasone group than in the placebo group.

Although we included adults aged over 18 years, the mean age of all the participants in the included studies was over 60 years, except for one study [26]. It is likely that older patients are at higher risk of POCD and POD than younger patients; therefore, evidence related to the incidence of POCD and POD in younger patients (< 60 years old) is still lacking. All the studies recruited patients that were scheduled for cardiac surgery except for one [26], which included participants that underwent microvascular decompression; this may limit the applicability of the evidence. Consequently, we should be careful not to extrapolate its use to patients undergoing other types of surgery. However, when we excluded this study from the meta-analysis, we found that the direction of the evidence did not change. There are two ongoing studies that were found through the clinical trials registration platform that need to be followed-up once they are completed [20, 21]. They may change the conclusion of this meta-analysis.

Only one study was found to have a low risk of bias in all domains [22], whereas the other four studies had a high or unclear risk of bias in at least one of the seven domains [23–26]. Two studies did not register their clinical trials or have a published study protocol, so the risks of selective reporting bias were unclear [23, 26]. We used the GRADE framework to assess the quality of the evidence. We considered the quality of the evidence to be very low for the incidence of POCD because of inconsistencies in the results and imprecision of the result. The inconsistencies might be explained by the differences in the types of surgery performed, the dose of dexamethasone administered and the definitions and assessment tools used in the diagnosis of POCD. However, we found no reduction in heterogeneity when we conducted the subgroup analyses of cardiac surgery versus non-cardiac surgery and low-dose (≤0.2 mg/kg) versus high-dose (> 0.2 mg/kg) dexamethasone. We also deemed the quality of the evidence for the incidence of POD to be very low because (1) the two studies were at ‘unclear risk’ of reporting bias and (2) the result was imprecise. The result relating to the incidence of POD mainly came from one study (weight 98.4%) and has wide CI.

It is of note that three studies [22, 24, 26] used neuropsychological tests recommended in the Statement of Consensus on the Assessment of Neurobehavioral Outcomes after cardiac surgery to assess the incidence of POCD [28], which has proved to be of immense value in providing a sensitive means of assessing the change and in detecting beneficial results associated with specific interventions [29–31]. The incidence of POCD in the control group of our study is 19%. By using a battery of neuropsychological tests, Johnson [32] found that the incidence of POCD in those aged more than 60 years was 19.2%, which was comparable with our study. Mardani et al. used MMSE, and Sauer et al. utilised CAM-ICU to assess the occurrence of POD [23, 25]. Unfortunately, MMSE scores are not a very reliable diagnostic test of POD; the diagnostic sensitivity and specificity were 96 and 38%, respectively [33]. CAM-ICU had 93–100% sensitivities, 98–100% specificities and high interrater reliability in the detection of delirium [34]. The incidence of POD in the control group of our study was 13%. However, the reported incidence of POD varied widely depending on the clinical setting. A systematic review found that the incidence of POD was about 11–51%, and the incidence increased with age [35]; hence, it was difficult to compare the incidence of POD.

It was disappointing to find that dexamethasone could not reduce the occurrence of POCD and POD in our meta-analysis, particularly since increased studies have shown that inflammatory cytokines are associated with POCD and POD [10, 11, 36]. The reasons for this phenomenon may be as follows: (1) The occurrence of POCD and POD results from the interaction of many predisposing factors and susceptible factors [37], and a single intervention cannot fully influence the incidence of POCD and POD. (2) Although there is some evidence that inflammation is an important mechanism for POCD and POD, other factors that have not been identified might play a greater role in the development of POCD and POD.

Orena et al. performed a systematic review to explore the effect of anaesthesia on POD and found that dexamethasone may reduce the risk of POD [38]. However, dexamethasone did not reduce the incidence of POD in our meta-analysis. The reason for this difference was that Orena et al. only included one study [23], in which the results of the first post-operative day exhibited a significant reduction of delirium incidence in the dexamethasone group compared with the control group, but not in the second and third post-operative days. However, the number of patients included in this study was small (43 in the dexamethasone group and 50 in the control group), and the MMSE was used to evaluate the occurrence of POD, which is not a very reliable diagnostic test of POD, and the specificity was just 38%. Toner et al. assessed the safety of glucocorticoids in non-cardiac surgery patients and found no increase in the risk of infection, a clinically unimportant increase in the glucose value and a lower concentration of CRP, but there was no difference in the length of hospitalisation [39]. In our meta-analysis, only one study reported the level of CRP and found it to be lower in the dexamethasone group compared with the placebo group (*P* < 0.001). Two studies reported the length of hospitalisation (measured in days), which revealed that the use of dexamethasone reduced the length of hospital stay (WMD − 0.57 days; 95% CI [− 1.08, − 0.07], *P* = 0.03, *I*^2^ = 0%), but the difference was so small that it did not have a clinical significance.

There are some limitations to this study. Firstly, we conducted a comprehensive search of the effect of dexamethasone on POCD and POD in several databases. However, these conclusions need to be interpreted with caution because of the limited number of evidence and the influence of the heterogeneity, especially the existing clinical heterogeneity, such as the type of surgery, the time and dose of dexamethasone administered and the different definition and evaluation tools of POCD and POD, which may limit the precision and reliability of the results. Secondly, since the incidence of POCD and POD was our primary outcome, randomised clinical trials that did not contain POCD and POD data were excluded. These studies may evaluate the secondary outcomes; thus, our meta-analysis of secondary outcomes raises no claim to completeness. Most studies excluded patients at high risk of POCD and POD; therefore, the application of conclusions may be limited.

## Conclusion

Our meta-analysis is the first systematic review to confirm the effects of dexamethasone on the incidence of POCD and POD in adults following general anaesthesia. This meta-analysis revealed that prophylactic dexamethasone did not reduce the incidence of POCD and POD. Trials of alternative preventive strategies for POCD and POD, as well as a better understanding of the pathophysiology of those complex syndromes, are still needed to make progress in this field.

## Additional file

Additional file 1: **Appendix 1.** MEDLINE (OvidSP) search strategy. **Appendix 2.** CENTRAL search strategy. **Appendix 3.** Embase(OvidSP) search strategy. (DOC 24 kb)

## Data Availability

All data generated or analysed during this study are included in this published article.

## Abbreviations

CABG: Coronary artery bypass graft
CAM: Confusion Assessment Method
CAM-ICU: Confusion Assessment Method adapted for the ICU
CI: Confidence intervals
CRP: C-reactive protein
GRADE: Grading of Recommendations, Assessment, Development and Evaluations
ICU: Intensive care unit
MMSE: Mini-Mental State Examination
POCD: Post-operative cognitive dysfunction
POD: Post-operative delirium
PRISMA: Preferred Reporting Items for Systematic Reviews and Meta-Analyses
RR: Relative risk
WMD: Weighted mean differences
