# Supplementary material for: Effects of dexamethasone on post-operative cognitive dysfunction and delirium in adults following general anaesthesia: a meta-analysis of randomised controlled trials
Source: BMC Anesthesiol. 2019 Jun 29;19:113. doi: 10.1186/s12871-019-0783-x (PMC6599229; doi:10.1186/s12871-019-0783-x)
Supplement: Supplementary file 1 — Appendix 1. MEDLINE (OvidSP) search strategy. Appendix 2. CENTRAL search strategy. Appendix 3. Embase(OvidSP) search strategy. (DOC 24 kb) [file 12871_2019_783_MOESM1_ESM.doc]

### Appendix 1.MEDLINE (OvidSP) search strategy

1. exp Dexamethasone/ or dexamethasone.ti,ab.
2. Anesthesia-and-Analgesia/ or Anesthesia/ or exp Anesthetics General/ or exp Anesthesia General/ or Postoperative Period/ or an?esth*.ti,ab.
3. Surgery/ or (surgery or surgical*).ti,ab.
4. 2 or 3
5. ((randomized controlled trial or controlled clinical trial).pt. or randomized.ab. or placebo.ab. or clinical trials as topic.sh. or randomly.ab. or trial.ti.) not (animals not (humans and animals)).sh.
6. 1 and 4 and 5

### Appendix 2. CENTRAL search strategy

#1 MeSH descriptor: [Dexamethasone] explode all trees

#2 dexamethasone:ti,ab,kw (Word variations have been searched)

#3 #1 or #2

#4 MeSH descriptor: [Anesthesia and Analgesia] explode all trees
#5 MeSH descriptor: [Postoperative Period] explode all trees
#6 (anaesth* or anesth*):ti,ab

#7 MeSH descriptor: [General Surgery] explode all trees

#8 (surgery or surgical):ab
#9 #4 or #5 or #6 or #7 or #8

#10 #3 and #9

### Appendix 3. Embase(OvidSP) search strategy

1 'dexamethasone'/exp OR dexamethasone.ti,ab

2 anaesthesia/ or exp general anaesthesia/ or an?esth*.ti,ab or postoperative period/

3 Surgery/ or (surgery or surgical*).ti,ab.

4 2 or 3

5 (placebo.sh. or controlled study.ab. or random*.ti,ab. or trial*.ti,ab. or ((singl* or doubl* or trebl* or tripl*) adj3 (blind* or mask*)).ti,ab.) not (animal not (human and animal)).sh.

6  1 AND 4 AND 5
